# Strengths, weaknesses, opportunities, and threats (SWOT) of the electronic prescribing systems executed in Iran from the physician’s viewpoint: a qualitative study

**DOI:** 10.1186/s12911-024-02687-w

**Published:** 2024-09-30

**Authors:** Mohamad Jebraeily, Shahryar Naji, Aynaz Nourani

**Affiliations:** 1 Health and Biomedical Informatics Research Center, Urmia University of Medical Sciences, Urmia, Iran; 2 Department of Health Information Technology, School of Allied Medical Sciences, Urmia University of Medical Sciences, Urmia, Iran; 3 Student Research Committee, Urmia University of Medical Sciences, Urmia, Iran

**Keywords:** Electronic prescribing, Information system, Technology, Qualitative research

## Abstract

**Background:**

Electronic prescribing (e-prescribing) is an essential technology in the modern health system. This technology has made many changes in the prescription process, which have advantages and disadvantages and have created opportunities for transforming the health system. This study aimed to investigate the strengths, weaknesses, opportunities, and threats of the e-prescribing system implemented in Iran from the physician’s viewpoint.

**Methods:**

This phenomenological qualitative study was conducted in 2022. The participants were 15 Iranian specialist physicians working at Urmia University of Medical Sciences, selected purposively and deliberately. Data was collected through in-depth semi-structured interviews using an interview guide comprising 16 questions. Interviews were conducted until data saturation was reached. The audio data was transcribed into text and analyzed using the thematic analysis. To ensure the validity and reliability of the findings, the criteria introduced by Lincoln and Guba were employed.

**Results:**

The results of this study showed that the e-prescribing system executed in Iran has diverse and multidimensional strengths, weaknesses, opportunities, and threats. In the strengths section, the analysis of the interviews led to the extraction of semantic units in the categories of prescription process, prescriber, patient, technical, economic, communication, and insurance. Also, the weaknesses in the three categories of the prescriber, patient, and technical were debatable. The opportunities extracted from the narratives of the interviewees were placed in four categories including technical, national macro policies, Ministry of Health macro-policies, and socio-cultural issues. Finally, the discussed threats are classified into two technical and macro policies of the Ministry of Health categories. On the other hand, technical issues played an effective role in all aspects of the SWOT model.

**Conclusion:**

The e-prescribing system in Iran has strengths, weaknesses, opportunities, and threats. An overarching factor across all aspects of the SWOT model was technical infrastructure. A robust technical infrastructure is considered a strength and an opportunity for the growth of the electronic prescribing system in Iran. Conversely, any shortcomings in these systems are viewed as weaknesses and pose a threat to the system’s sustainability.

**Supplementary Information:**

The online version contains supplementary material available at 10.1186/s12911-024-02687-w.

## Background

Over the years, handwritten prescriptions have been the preferred method of communication between physicians and other healthcare professionals in documenting and providing medical orders [1]. Pharmacies, laboratories, medical imaging centers, and other parts of the health system use paper prescriptions to provide services and execute orders [2]. Despite its simplicity and ease of implementation, manual prescription brings many problems [3]. Problems related to patient safety, medical errors, abuse of health services and drugs, and waste of resources are among the most common disadvantages caused by manual prescribing. These can defeat the main goal of the healthcare system, which is to provide effective, safe, and useful health services for patients [4]. Due to the defects and disadvantages, the need for change and evolution in manual prescriptions has always been the focus of different countries [5]. For this reason, when information technology entered the healthcare industry, one process affected by technology was the prescription process [3].

Electronic prescribing (e-prescribing) is a technological solution that can facilitate the prescribing process and solve many related problems [2]. E-prescribing systems are different in terms of capabilities and facilities; however, such a system at least includes a software interface to document orders and a communication system to send the prescription to the destination. Today, Decision Support Systems (DSSs) and Electronic Health Records (EHRs) are an integral part of the e-prescribing system. DSSs provide real-time clinical decision support, while EHRs enable comprehensive patient data management, both of which bring significant benefits to the e-prescribing process [6, 7]. However, the results of the studies indicate that e-prescribing systems have not been successful in some cases, and contrary to the expectations of developers and stockholders, these have caused problems or exacerbated existing problems [8].

Various sociotechnical factors, organizational culture, users’ attitudes, and organizational and extra-organizational policies and strategies can influence the entire implementation process and, ultimately, the success and failure of the e-prescribing system [9]. Factors related to project management, such as the lack of stakeholder participation, lack of system maturity, and insufficient performance, can directly lead to a superficial understanding of the system’s benefits and lack of integration in the clinical workflow. Therefore, acceptance and success of the e-prescribing system depend on the detailed investigation and analysis of the e-prescribing from different dimensions and at different stages of the system’s life cycle [10].

Different methods exist for investigating and analyzing e-prescribing systems and other information systems [11]. The Stanford Research Institute developed and used SWOT analysis, which investigates Strengths, Weaknesses, Opportunities, and Threats, for the first time. SWOT analysis is one of the strategic management methods of organizations that can help decision-making [12]. This method has been successfully executed in health information technologies to investigate the strengths, weaknesses, opportunities, and threats of EHRs [13], telemedicine [14], Etc. However, there are few studies related to its use to investigate e-prescribing systems.

In Iran, a developing country, e-prescribing in hospitals has been used since 2019. According to the technology acceptance model, all new technologies, especially in developing countries, must go through a path with many opportunities and threats [15]. Like any other technology, the success and failure of e-prescribing require identifying strengths and weaknesses, opportunities and threats, and planning to manage these factors. Despite the importance of examining the prescription system from these four dimensions, no similar study has been conducted in Iran. However, the SWOT method has yielded positive results in studies analyzing electronic prescribing systems in other countries [16, 17]. For example, in Germany, the SWOT model was employed to assess stakeholder analysis of electronic prescribing [16]. Similarly, a study in Thailand utilized the SWOT method to develop a protocol for an electronic prescription system for government sector outpatients and private drug stores [17].

However, regarding the evaluation of e-prescribing systems, limited studies have been conducted in Iran. In a study conducted in 2022, the user interface of two main e-prescribing systems was evaluated. The results indicated that the user interface of both systems is in an average condition in terms of usability [18]. In another study conducted in 2023, patients’ perceptions, experience, and satisfaction with the e-prescribing system were investigated. The results showed that most of the patients demonstrated awareness of the e-prescribing system and expressed a preference for electronic prescribing. Patients reported overall positive satisfaction and relatively positive perceptions and experiences with the evaluated e-prescribing system [19].

Conducting the present study was important in several aspects. First, in the agenda of the Iranian Ministry of Health, the e-prescribing systems have a special place, and this Ministry is committed to achieving its goals (improving health services) by supporting the existing systems and implementing them at the national level. However, these systems should be investigated through various studies. In this regard, examining the strengths, weaknesses, opportunities, and threats of e-prescribing systems can be effective in the Ministry of Health’s policymaking to strengthen this system. Second, few studies have been conducted on evaluating electronic prescribing systems in Iran, especially from the perspective of its main users [3, 20–23]. Therefore, it seems necessary to conduct studies that can identify the strengths and weaknesses of the system and, on the other hand, opportunities and threats to prepare for future directions, deal with threats, and strengthen opportunities. Thirdly, most of the related studies carried out in Iran were quantitative [18, 19, 21]. With the limitations of the data collection tools, it was difficult to capture the diverse perspectives of the research community and present comprehensive and detailed information. Therefore, the present study was conducted to investigate and analyze the main strengths, weaknesses, opportunities, and threats of the e-prescribing system executed in Iran from the users’ viewpoint through in-depth interviews with a qualitative approach.

## Methods

The present phenomenological qualitative study was conducted in 2022. The participants were 15 physicians working in hospitals affiliated with the Urmia University of Medical Sciences, who were selected using the purposive sampling method. The inclusion criteria included having at least six months of experience working with the e-prescribing system, having a personal interest in electronic health services, and having enough time for in-depth discussion within the framework of the present study. The exclusion criteria were unwillingness to continue the interview.

The interviews were conducted using an in-depth inquiry approach and a semi-structured interview guide (supplementary file 1). The interview guide was prepared by reviewing the literature [12, 13, 24, 25] and included four closed questions about the interviewee’s background information and 12 open questions about the implemented e-prescribing system. The content validity of the interview guide was confirmed by two specialists in medical informatics and health information management. These two specialists were selected by the easy access method from medical informatics and health information management faculty members of Urmia University of Medical Sciences. They had a history of research on e-prescribing systems and were aware of the different dimensions of these systems and their evaluation methods.

To obtain suitable data and the approximate time needed to interview, two interviews were conducted on a trial basis, and the interview guide was modified based on their analysis. Finally, these two interviews were not considered in the final analysis. After obtaining written informed consent, the interviews were conducted in the presence of the participants and the interviewer at the specified place (physicians’ office) and time (at the end of working hours). All interviews were conducted face-to-face. The interviews continued until data saturation. In this study, after conducting 13 interviews, data saturation seemed to have occurred. However, two more interviews were conducted to make sure.

The current research employed a thematic analysis technique, following a five-step process of data analysis. This approach involved becoming familiar with the data, creating initial codes, reviewing the codes, identifying and naming the categories, putting categories in the SWOT framework, and finally generating a report based on the findings. Coding and data analysis were done in the framework of four main themes in Atlas.ti software. The criteria introduced by Lincoln and Guba were used to ensure the validity and reliability of the findings [26]. Therefore, the findings’ credibility, transferability, reliability, and confirmability were checked.

Credibility was confirmed by assigning sufficient time to collect data until data saturation and three participants’ independent confirmation of study findings. In addition, to obtain the necessary and credible data, the researchers established a suitable social relationship with the interviewees, and sampling was done with the highest variety of participants. This means that an effort was made to select those different in medical specialties, age, and gender among the eligible cases to participate in the study. Reliability was guaranteed through the participation of three researchers in the same analysis to provide different perspectives, adding breadth to the study phenomenon and multiple conclusions. Also, the researchers collected and analyzed the data simultaneously and continuously and ignored any initial assumptions about the findings. Conformability was assured by discussing the codes, subthemes, and themes with the research team and participants of the study to obtain consensus. Finally, two expert faculty members approved the method of coding and obtaining sub-themes and categories in qualitative research. Finally, transferability was observed by confirming the findings of three physicians (eligible to participate in the study) who were not among the participants.

## Results

In this qualitative study, 15 physicians working in public hospitals affiliated with the Urmia University of Medical Sciences participated. The average duration of the interviews was 57 min. Table 1 shows the demographic information of the participants.

According to Table 1, most participants were male (*n* = 10, 66.67%) and had worked with the e-prescribing system for more than six months. Also, more than half of the participants (*n* = 11, 73.33%) were 45–55 years old.

**Table 1:** Demographic characterization of study participants

Variable		Frequency (Percentage)
Sex	Male	10 (66.67)
	Female	5 (33.33)
Age	35–45	4 (26.67)
	45–55	11 (73.33)
Specialty	Nephrology	1 (6.67)
	Surgery	2 (13.33)
	Neurology	3 (20.00)
	Lung and respiratory	1 (6.67)
	Digestion and liver	2 (13.33)
	Internal medicine	2 (13.33)
	Urology	1 (6.67)
	Cardiology	2 (13.33)
	Obstetrics and Gynecology	1 (6.67)
Work experience with the system	> 1year	3 (20.00)
	< 1year	12 (80.00)

**Fig. 1:**
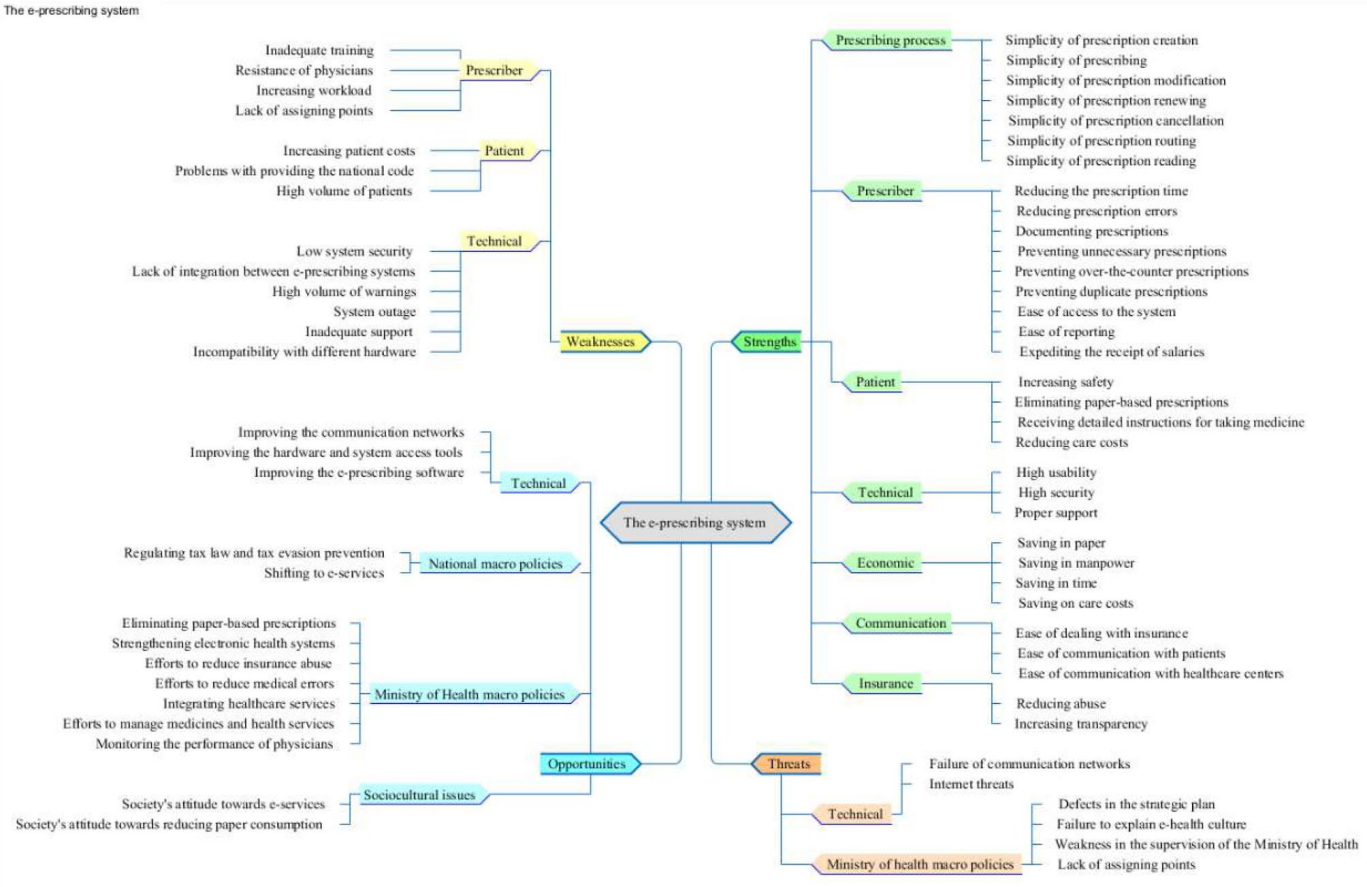
Themes, sub-themes, and categories

The analysis of the participants’ viewpoints regarding the strengths, weaknesses, opportunities, and threats of the e-prescribing system led to the extraction of 187 semantic units. After examination and integration, semantic units were organized into four themes and 16 sub-themes (Fig. 1). In qualitative studies, the structure of themes, sub-themes, and categories is formed based on the interviewees’ statements. In this study, we tried to provide the most rational structure with the least overlap by examining and correctly integrating and separating the semantic units.

## Strengths

The results showed that the strengths of the executed e-prescribing systems could be divided into seven categories: “prescribing process,” “prescriber,” “patient,” “technical,” “economic,” “communication,” and “insurance.”

In the “prescription process” sub-theme, the interviewees acknowledged that e-prescribing makes the prescription process simple and easy, from when the prescription is created to when the orders are executed. Therefore, one of the strengths of the e-prescribing system is the simplicity of creating a prescription, prescribing, modifying, renewing, canceling, routing the prescription, and reading the prescription. In this regard, one of the interviewees stated: “E-prescribing has been able to greatly facilitate the correction, cancellation, or even renewal of the prescription.” (Participator 1).

The second sub-theme was prescriber. Most interviewees believed that the e-prescribing system had many benefits for them. So, their time and material value are preserved by reducing the prescription time, speeding up the receipt of salaries, reducing the burden of their professional responsibilities, documenting prescriptions, and preventing unnecessary and repetitive prescriptions. They also mentioned that the e-prescribing system is accessible from any geographical location and with any hardware, and they can easily access the system to prescribe and report their activities. In this regard, one of the interviewees pointed out: “This system is transparent, and there is less chance of wrong prescription of drugs. If a drug is given to a patient by mistake, it will be whether the pharmacy or the physician made the mistake.” (Participator 4).

Patients are another stakeholder of e-prescribing systems. The conversations of the interviewees indicated that the executed e-prescribing systems have strengths that benefit the patient. They pointed out that increasing the safety of patients, not having to carry paper-based prescriptions, receiving accurate medication orders, and reducing care costs are the most important benefits of e-prescribing for patients. In this regard, one of the interviewees pointed out: “With e-prescribing, there is continuity of proper care, and the patient is not referred for a CT scan twice in one week, because the previous documents are available in the system. Therefore, the insurance and the patient do not suffer financial losses.” (Participator 7).

Another sub-theme in this theme was technical strengths. Some interviewees admitted that the executed e-prescribing systems are usable and can provide a suitable platform for the electronic execution of the prescribing process by maintaining sufficient security and support.

Economic strengths were another sub-theme in this theme. The information provided by the interviewees indicated that e-prescribing reduces the economic burden of the health system by saving paper, manpower, physicians’ time, and care costs. In this regard, one of the interviewees pointed out: “All the costs of paper, print, and printer are our capitals, which can be saved by e-prescribing”. (Participator 5)

Another strength of e-prescribing was facilitating communication. Some interviewees pointed out that the executed e-prescribing systems have facilitated communication with patients, insurance companies, and healthcare centers by creating a suitable infrastructure. In this regard, one of the interviewees said: “It is easier to follow patients and establish a relationship with them in the system. All patient information can be accessed with the national number.”

The final sub-theme was Insurance. The interviewees believed that e-prescription has reduced the abuse of insurance and increased transparency in this regard. One of the interviewees mentioned, “This system monitors prescriptions and as a result, while increasing transparency in services including insurance as well as the number of these services, it eliminates the possibility of abusing insurance for both the patient and the physicians.” (Participant 13).

## Weakness

In general, the weaknesses mentioned by the interviewees were debatable in the three sub-themes including “prescriber”, “patient” and “technical”.

Regarding the weaknesses related to the prescriber, the interviewees mentioned inadequate training, the resistance of physicians, increasing workload, and lack of assigning points. Some interviewees believed physicians have less time to train computer skills and use the system due to clinical workload. They admitted that without adequate training and by trial and error, it is impossible to provide a correct prescription, and ultimately, this will cause incorrect prescriptions and create risks for the patient’s safety. The content of the interviews showed that physicians resist using the system for various reasons. They considered one of the reasons for physicians’ resistance to be not receiving appropriate financial benefits and lack of sufficient training. In this regard, one of the physicians pointed out: “Working with the system has increased my workload, it takes me a lot of time to search for drugs, prescribe them one by one, adjust the dosage and how to take the drugs.” (Participator 10).

Another weakness of the system was related to the patient. The interviewees considered the increasing patient costs, the problems of presenting the national number, and the high volume of patients as issues related to the patient. Some interviewees believed that elderly patients face problems in providing the national number, and the executed e-prescribing systems cannot provide a prescription without the national number. Also, the interviewees considered the increasing patient costs to be one of the weak points of using the e-prescribing system. They stated that some healthcare services are unavailable in the system and cannot be prescribed, and the patient should pay for them freely. In this regard, one of the interviewees pointed out, “… to put it simply, if you want to request a prostate ultrasound in these systems, there is no such thing, and we have to prescribe an ultrasound of the entire abdomen and pelvis. This imposes additional costs on the patient.” (Participator 6).

Another weakness of the executed e-prescribing system was related to technical issues. The interviewees mentioned low system security, lack of integration between e-prescribing systems, high volume of warnings, system outages, inadequate support, and incompatibility with different hardware as the main technical weaknesses of the system. Most of the interviewees believed that due to the lack of electronic signatures, the access of secretaries and other employees to physicians’ usernames and passwords, the lack of appropriate authentication methods, and other issues, the security of the e-prescribing system is insufficient. In this regard, one of the interviewees pointed out: “Because of the lack of electronic signature, the security is very low, and I can even see the patients of other physicians and write prescriptions for them.” (Participator 2).

Also, the interviewees were dissatisfied with the lack of integration between e-prescribing systems. Because they must use different systems based on the type of patient insurance. Most of the physicians were dissatisfied with the system’s high volume of warnings when prescribing the drug and entering the drug dose. Outages and fluctuations in electricity and communication networks and weak support of the development companies from system problems were also other weaknesses that caused physician dissatisfaction.

## Opportunities

In the SWOT method, opportunities refer to all factors outside the system that can positively affect its success [27]. The current study organized the opportunities raised by the interviewees into four categories: “Technical,” “National macro-policies,” “Ministry of Health macro policies,” and “Sociocultural.”

Technical issues were one of the issues emphasized in the success of the e-prescribing systems. The interviewees believed that rapid advances in technology, both in terms of the Internet and communication networks and in terms of hardware, can be a turning point in the success of e-prescribing. Also, the interviewees believed that along with the growth of hardware and communication networks, e-prescribing software has also experienced significant improvement. In this regard, one of the interviewees pointed out:” Indeed, the development of technology, both hardware and software, accelerates the acceptance of this system.” (Participator 7).

Also, the interviewees brought up opportunities that were part of the macro policies of Iran country and the government and played a role in advancing e-prescribing. One of these cases was regulating tax laws and government strategies to reduce tax evasion by citizens, including physicians. They believed that e-prescribing gives you a clearer picture of physicians’ performance. Therefore, the support of the government and the Ministry of Health can be an engine for the progress and success of e-prescribing systems. On the other hand, most physicians pointed to the necessity of the new era and the government’s policy in moving towards e-services. They acknowledged that the country’s policies for establishing electronic government could be a potential for implementing e-health. Physicians believe that e-prescribing is an important part of e-health.

About the opportunities for the growth and success of the executed e-prescribing systems, the interviewees pointed to the policies of the Ministry of Health to eliminate paper-based prescribing, strengthen electronic health systems, try to reduce medical errors and misuse of insurance, monitor the performance of physicians, integrate health service delivery system, and medication management. They believed that removing the paper-based prescription all at once, although the physicians did not accept it, was able to force them to use the system. Also, the interviewees pointed out that strengthening health systems and the plan that the Ministry of Health has to advance them can lead to the development of e-prescribing systems by creating an electronic health culture. On the other hand, the interviewees emphasized the Ministry’s effort to monitor physicians’ performance, reduce insurance abuse, and manage medications and services that e-prescribing can achieve. Regarding the management of medications and services, considering the limited resources in Iran, one of the policies of the Ministry of Health was to support e-prescribing systems to prescribe medications and diagnostic services rationally. In this regard, one of the interviewees said: “There are drug restrictions and its management. Despite e-prescribing and with the help of warnings from this system, prescriptions are done correctly. Violations have decreased, and drug management has improved. These advantages have made e-prescribing more accepted in society.” (Participator 14).

Finally, interviewees pointed to socio-cultural issues as an opportunity to enhance e-prescribing. Some of the interviewees pointed out that there is a positive collective attitude towards electronic services, and most people want to receive their services electronically from all public and private organizations. This attitude can strengthen e-prescribing. In this regard, one of the interviewees said, “Currently, we are in a period where negative attitudes and resistance to e-services have disappeared and have been replaced by positive views. These positive attitudes are an opportunity to grow and strengthen e-prescribing.” (Participant 10) Also, nowadays, the culture of saving paper and reducing its use to protect forests has become established. People considered paper prescriptions, which were prescribed in large quantities daily, to be wasteful. Some interviewees mentioned that this culture of reducing paper consumption could be an opportunity to accept e-prescribing.

## Threats

Threats refer to factors outside the system that can contribute to its failure [27]. In general, the threats raised by the interviewees were debatable in the two sub-themes of technical threats and Ministry of Health macro policies.

Although the interviewees evaluated the state of hardware and communication networks at a suitable level compared to previous years, however, they were not satisfied enough with the communication infrastructure and the Internet. On the other hand, they considered cyber threats as a threat to e-prescribing, which is happening on the Internet. In this regard, one of the interviewees pointed out, “I am not satisfied with the internet situation. The internet may be interrupted for various economic and social reasons, or even the existing system may be attacked by cyber. Therefore, we cannot be 100% dependent on it.” (Participant 15).

On the other hand, the interviewees mentioned threats that were all related to the macro policies of the Ministry of Health. Issues such as defects in the strategic program of e-prescribing, failure to explain the culture of e-health, weakness in surveillance, and lack of assigning privileges to physicians were the threats raised in this regard. Some interviewees believed that e-prescribing does not have a systematic strategic plan and that the Ministry of Health acted hastily in its implementation without considering physicians’ preparation and working conditions. They stated that the e-health culture is not yet dominant in Iran, so accepting e-prescribing for all stakeholders, including patients, providers, and insurance companies, is associated with challenges. These challenges can be a threat to the system. On the other hand, the theme of the interviews was that the most important threat to the e-prescribing system is the resistance and dissatisfaction of physicians. They stated that the relevant Ministry did not assign any privileges to physicians; for this reason, they consider e-prescribing a kind of imposition and have a negative position against it.

## Discussion

In this study, the viewpoints of specialist physicians as the main users of the e-prescribing system were collected and analyzed. The results showed that physicians, while aware of the generalities of the executed e-prescribing systems from different dimensions, can review the system’s strengths, weaknesses, opportunities, and threats. Among the items raised in the SWOT framework, physicians discussed the system’s strengths more than other dimensions.

The results of the present study showed that the e-prescribing systems executed in Iran have provided many strengths, including facilitating the prescription process, reducing the prescriber’s workload and professional responsibilities, and increasing patient satisfaction. Also, these systems have brought benefits to the country’s health system in terms of technical, economic, communication, and insurance. In the study of Miller et al., the prescription process is defined as a process in which the e-prescribing system can be effective from various sociotechnical aspects in the prescription process and communication between the prescriber, the order executor, and the patient [28]. Also, Griffon et al.‘s study results are in line with the present study’s results and declare the strengths of e-prescribing in improving the health system’s economic situation and the communication situation for the parties participating in the prescription process [29]. The results of Lyons et al.‘s study, while confirming the results of the present study, point to the role of e-prescribing in reducing repeated tests and reducing the length of hospitalization of patients [30].

Regarding the weaknesses of e-prescribing, the results of the current study indicated that the execution of this system in Iran was not technically correct and caused problems. Also, in some cases, the system is not cost-effective for patients, and due to the crowding of patients in public medical centers and the problems of providing national numbers, it has caused disruptions in the prescription process. Physicians, as the system’s main users, express their dissatisfaction with weak points such as insufficient training, increased workload, and lack of receiving special points and resist it. The results of the study by Kruse et al. showed that the problems of training physicians and resistance to changing the current process of prescribing can cause the failure of e-prescribing [31]. Therefore, the results of the present study align with the results of Kruse’s study. However, in the study of Cross, the reduction of productivity (disruption in the normal process of doing work) has been mentioned as one of the weak points of e-prescribing. In the present study, the interviewees implicitly mentioned the disruption of work and the change in the common process. In particular, the weak points of the e-prescription system, as in the present study, in the study of Vermeulen et al., technical problems and system errors in increasing the costs of patients (from 12.37 to 14.91 euros) have been pointed out, which is in line with the results of the present study [6].

The findings of the present study indicated that the upward trend in the development of hardware, communication networks, and e-prescribing software could be an opportunity to advance this system in Iran. While emphasizing the role of the necessary equipment to execute the system, Holden considered the prescription software quality as an opportunity to encourage users [32]. Also, the results of the present study showed that the national macro policies and the macro policies of the Ministry of Health could be an opportunity for the acceptance and growth of e-prescribing in Iran. In other studies, it has been pointed out that the support and policies of the government and information technology are aligned. Zadeh et al. have emphasized the national pressures and the role of policies such as monitoring physicians’ performance in advancing the goals of e-prescribing [33]. Finally, the results of the present study showed that a positive attitude towards saving paper, which is one of the national resources, as well as a positive attitude towards e-services, can be an opportunity for e-prescribing. This topic has also been considered in other studies [34, 35]. Alipour et al. considered saving paper as one of the advantages of electronic prescribing [34].

The results of the present study showed that technical problems and internet and communication network defects can significantly threaten the system’s success. Zadeh et al. have discussed technical threats and their impact on the failure of e-prescribing [33]. Also, the results of the present study indicated the role of the Ministry of Health’s macro policies, such as the lack of explanation of e-health culture and weakness in monitoring and strategic plan, in the success of e-prescribing. Weakness in surveillance and superior organizations and the lack of alignment of their policies and plans with information technology can threaten the success of any information system, including e-prescribing [2].

## Conclusion

The e-prescribing system in Iran, supported by the Ministry of Health, exhibits strengths and weaknesses. The technical infrastructure plays a pivotal role, serving as both an advantage and a limitation. Strengthening this infrastructure presents an opportunity for system enhancement, while neglecting it poses a threat to electronic prescribing sustainability in the country. The results of this study can be useful for other developing countries that want to implement an e-prescribing system or evaluate their current e-prescribing system. The strengths and weaknesses presented in this study, expressed from the viewpoint of the system’s main users, can help design new systems or improve current e-prescribing systems. Also, the mentioned threats and opportunities can be used as a guide to identify opportunities and threats and plan to deal with threats or strengthen opportunities.

## Research limitations

At the time of conducting this study, due to several reasons, such as the long geographical distance from the northwest of the country to its center and the prevalence of the Omicron strain of COVID-19, it was not possible to travel to the Ministry of Health and interview with decision-makers in e-prescribing area. The researchers tried to interview the decision-makers in this area through video conference or phone calls. But unfortunately, they did not have enough time to interview and participate in the study. However, to obtain the maximum necessary and possible data, we tried to interview physicians who, in addition to their experience working with the system, are also connected with policymakers in this field. Therefore, among the interviewees, two physicians have participated several times in the important meetings on e-prescribing that have been held in the form of video conferences in the country.

## Electronic supplementary material

Below is the link to the electronic supplementary material.

Supplementary Material 1: Supplementary file 1 Interview guide

## Data Availability

The data supporting this study’s findings are available on request from the corresponding author. The interview files are not publicly available due to restrictions e.g., their containing information that could compromise the privacy of research participants.

## Abbreviations

e-prescribing: Electronic-prescribing
EHR: Electronic Health Record
CDSS: Clinical Decision Support System
SWOT: Strengths, Weaknesses, Opportunities, and Threats
