# Supplementary material for: Strengths, weaknesses, opportunities, and threats (SWOT) of the electronic prescribing systems executed in Iran from the physician’s viewpoint: a qualitative study
Source: BMC Med Inform Decis Mak. 2024 Sep 30;24:279. doi: 10.1186/s12911-024-02687-w (PMC11441130; doi:10.1186/s12911-024-02687-w)
Supplement: Supplementary file 1 — Supplementary Material 1: Supplementary file 1 Interview guide [file 12911_2024_2687_MOESM1_ESM.docx]

**Supplementary file 1. Interview guide**

| Age: | ………………… | Specialty: | ………………… |
| --- | --- | --- | --- |
| Sex: | ………………… | Work experience with the system: | ………………… |

Question 1: In your opinion, what are the strengths of the electronic prescription system?

Question 2: In general, what benefits can the use of this system bring?

Question 3: In your opinion, what are the weaknesses of the electronic prescription system?

Question 4: In your opinion, what are the weaknesses of the electronic prescription system that will bring problems and issues?

Question 5: What is your suggestion to overcome the weaknesses of the electronic prescription system?

Question 6: In your opinion, what opportunities are there within the organization to advance the goals of electronic prescribing?

Question 7: In your opinion, what external opportunities are there to advance the goals of electronic prescribing?

Question 8: What is your suggestion for using these opportunities?

Question 9: In your opinion, what are the external threats to electronic prescribing?

Question 10: In your opinion, what are the internal threats to electronic prescribing?

Question 11: What is your suggestion to overcome these threats?

Question 12: Please state your general opinion regarding the prospect of electronic prescribing.
